# Activity-Dependent Modulation of Tonic GABA Currents by Endocannabinoids in *Hirudo verbana*

**DOI:** 10.3389/fnsyn.2022.760330

**Published:** 2022-03-14

**Authors:** Riley T. Paulsen, Brian D. Burrell

**Affiliations:** Division of Basic Biomedical Sciences, Center for Brain and Behavior Research, Sanford School of Medicine, University of South Dakota, Vermillion, SD, United States

**Keywords:** endocannabinoids, 2-arachidonoylglycerol (2-AG), anandamide (AEA), TRPV, tonic inhibition, *Hirudo verbana*, synaptic depression

## Abstract

Endocannabinoids are lipid neuromodulators that are synthesized on demand and primarily signal in a retrograde manner to elicit depression of excitatory and inhibitory synapses. Despite the considerable interest in their potential analgesic effects, there is evidence that endocannabinoids can have both pro-nociceptive and anti-nociceptive effects. The mechanisms contributing to the opposing effects of endocannabinoids in nociception need to be better understood before cannabinoid-based therapies can be effectively utilized to treat pain. Using the medicinal leech, *Hirudo verbana*, this work investigates whether endocannabinoids modulate tonic inhibition onto non-nociceptive afferents. In voltage clamp recordings, we analyzed changes in the tonic inhibition in pressure-sensitive (P) cells following pre-treatment with endocannabinoids, 2-arachidonoylglycerol (2-AG) or anandamide (AEA). We also tested whether high frequency stimulation (HFS) of nociceptive (N) cells could also modulate tonic inhibition. Both endocannabinoid application and N cell HFS depressed tonic inhibition in the P cell. Depression of tonic inhibition by N cell HFS was blocked by SB 366791 (a TRPV1 inhibitor). SB 366791 also prevented 2-AG-and AEA-induced depression of tonic inhibition. HFS-induced depression was not blocked by tetrahydrolipstatin (THL), which prevents 2-AG synthesis, nor AM 251 (a CB1 receptor inverse agonist). These results illustrate a novel activity-dependent modulation of tonic GABA currents that is mediated by endocannabinoid signaling and is likely to play an important role in sensitization of non-nociceptive afferent pathways.

## Introduction

Endocannabinoids are lipid neuromodulators synthesized in an activity-dependent manner (Castillo et al., 2012). The main endocannabinoid ligands are 2-arachidonoylglycerol (2-AG) and *N-*arachidonylethanolamide (AEA) and these can act on metabotropic cannabinoid receptors (CB1 and CB2) or transient receptor potential (TRP) channels, e.g., TRPV (Edwards, 2014; Muller et al., 2019; Ye et al., 2019). Synthesis takes place primarily in the postsynaptic cell, which allows them to function as retrograde messengers and modulate diverse forms of synaptic plasticity (Castillo et al., 2012).

Endocannabinoids can modulate excitatory or inhibitory synapses over both short- and long-term time scales, often via presynaptic depression of neurotransmitter release (Castillo et al., 2012). Furthermore, these effects can be homosynaptic or heterosynaptic (Piette et al., 2020). In situations where inhibitory synapses are depressed, this can lead to disinhibition of excitatory synapses (Zhu and Lovinger, 2007; Kim et al., 2019). This disinhibition effect can lead to endocannabinoids eliciting long-term potentiation (LTP) in excitatory, glutamatergic synapses (Carlson et al., 2002; Brown et al., 2013; Cui et al., 2015; Silva-Cruz et al., 2017). Endocannabinoid-mediated disinhibition and its effects on LTP are likely to play an important role in modulating nociceptive synaptic circuits. In rodents, while endocannabinoid-mediated depression of glutamatergic synapses in the spinal cord represents a likely anti-nociceptive effect (Kato et al., 2012), endocannabinoid depression of inhibitory synapses has also been observed and leads to disinhibition of nociceptive circuitry in the spinal cord that generates a pro-nociceptive effect (Pernia-Andrade et al., 2009).

There is interest in using comparative approaches to understand the basic biology of pain (Walters and Williams, 2019). Applying this approach to endocannabinoid modulation of nociception makes sense given the interest in cannabinoid-based approaches in treating pain and that the endocannabinoid system is well-conserved across the animal kingdom (Elphick, 2012; Paulsen and Burrell, 2019). The central nervous system (CNS) of the medicinal leech, *Hirudo verbana*, is an especially useful organism in which to carry out these studies. The *Hirudo* CNS possesses mechanosensory neurons that are functionally similar to mammals, including rapidly adapting touch (T), slow adapting pressure sensitive (P), and both mechanical and polymodal nociceptive (N) neurons (Nicholls and Baylor, 1968; Blackshaw et al., 1982; Pastor et al., 1996; Lewin and Moshourab, 2004; Abraira and Ginty, 2013; Burrell, 2017). Furthermore, recent studies have found that genes associated with mechanosensation in mammals are also expressed in the *Hirudo* mechanosensory neurons, e.g., piezo, DeG/ENaC, and trp encoding genes (Heath-Heckman et al., 2021). As in mammals, *Hirudo* possesses the endocannabinoids 2-AG and AEA, with the former being more abundant (Matias et al., 2001). *Hirudo* possesses genes encoding the proteins required for 2-AG synthesis, [diacylglycerol lipase (DAGL) α and β (accession #s KU500007 & MT610103), 2-AG metabolism (monoacylglycerol lipase (MAGL) (KY971276)], AEA metabolism [fatty acid amide hydrolase (FAAH) (pending)], and TRPV (Heath-Heckman et al., 2021). *Hirudo* MAGL (hirMAGL) has been studied in some detail and exhibits considerable structural and functional conservation at the protein’s catalytic active site (Kabeiseman et al., 2020). Using pharmacological approaches, multiple drugs blocking DAGL (RHC-80267, OMDM-188, and tetrahydrolipstatin/Orlisat) and activating (capsaicin, resiniferatoxin) or inhibiting (capsazepine, SB-366791) TRPV channels have been effective in studying putative endocannabinoid-mediated modulation of synapses and behavior in *Hirudo* (Yuan and Burrell, 2010, 2012, 2013b; Wang and Burrell, 2016, 2018).

The pro- and anti-nociceptive effects of endocannabinoids reported in rodent models (Pernia-Andrade et al., 2009; Kato et al., 2012; Sdrulla et al., 2015) are also observed in *Hirudo* (Yuan and Burrell, 2013b; Summers et al., 2017; Wang and Burrell, 2018). 2-AG activation of TRPV-like channels depresses nociceptive (N) cell synapses and decreases behavioral responses elicited by N cell activation (Yuan and Burrell, 2010, 2012, 2013b). On the other hand, endocannabinoids potentiate non-nociceptive pressure (P) cell synapses and increase the behavioral responses elicited by P cells (Higgins et al., 2013; Wang and Burrell, 2016, 2018). It is this latter effect that we will focus on.

**FIGURE 1:**
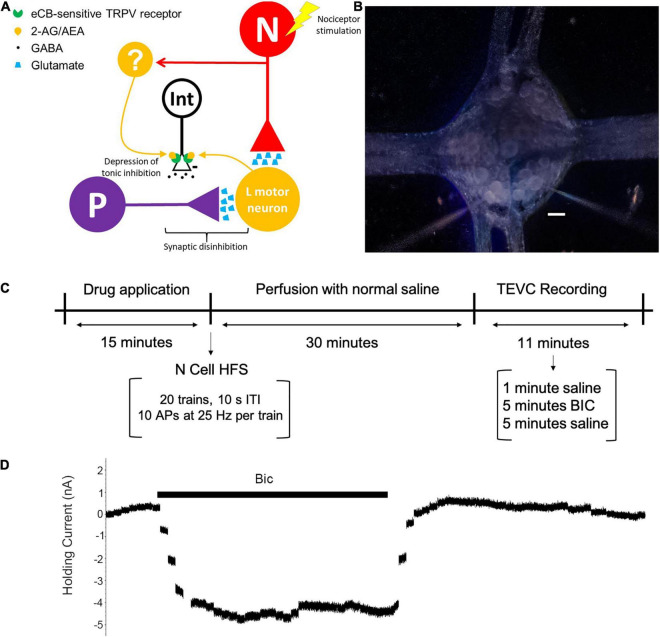
**(A)** Proposed circuit for N cell-initiated disinhibition of P cell synapses through activity-dependent endocannabinoid modulation. The N and P afferents have converging inputs onto multiple postsynaptic targets, including the longitudinal (L) motor neuron. An unidentified GABAergic interneuron (Int) has tonic inhibitory input onto the P cell [indicated by the minus (–) sign]. It is hypothesized that HFS of an N cell elicits endocannabinoid (2-AG or AEA) production that depresses synaptic transmission by the neuron that is the source of tonic inhibition on to the P cell. The source of endocannabinoid signaling may be the L motor neuron, which is known to produce 2-AG following low frequency afferent input (Yuan and Burrell, 2010). However, it is possible that other, unknown neurons (labeled as “?”) that also receive N cell input (indicated by the red arrow) are the source of 2-AG and/or AEA. **(B)** Acutely isolated ganglion with two microelectrodes. Scale bar = 50 μm. **(C)** Experimental timeline. Ganglia received a bath application of drug for 15 min followed by a 30 min wash-out period with normal saline. TEVC recordings (see section “Materials and Methods”) were next performed in the P cell by first perfusing with saline for 1 min to establish a baseline, followed by perfusion with BIC for 5 min, and 5 final minutes in normal saline in order to allow the cell to return to its baseline holding current. Control experiments without BIC were perfused with normal saline throughout the entire TEVC experiment. Experiments with N cell HFS received stimulation after a drug pretreatment (if applied) and before the washout period. **(D)** Example of BIC effect on holding current. Plot consists of a series of concatenated recordings of P cell holding current prior to, during, and after application of BIC (1 sec TEVC recording with 10 sec intervals between each recording over a period of 11 min).

Potentiation of P cell synapses by endocannabinoids is long-lasting (30–60 min) and proposed to be a disinhibition-mediated process, based on previous experiments in which inhibition of either GABA receptors or chloride importers prevents endocannabinoid-mediated potentiation (Higgins et al., 2013; Wang and Burrell, 2016). Disinhibition of P cell synapses is presynaptic in nature, supporting the idea that GABAergic input is modulating the amount of neurotransmitter (glutamate) released at the presynaptic terminal (Wang et al., 2015). In additional, high frequency stimulation (HFS) of the N cells also produces a similarly persistent potentiation of P cell synapses in *Hirudo* in a 2-AG-dependent manner that requires disinhibition (Wang and Burrell, 2016). Potentiation of P synapses by N cell HFS or exogenously applied endocannabinoids is TRPV-dependent, but P cells lack these TRPV-like channels (Higgins et al., 2013; Summers et al., 2014; Wang and Burrell, 2016). We hypothesize, as shown in Figure 1A, that endocannabinoids act on *Hirudo* TRPV-channels located on GABAergic neurons that modulate the P cell. The GABAergic neurons undergo endocannabinoid/TRPV-mediated long-term synaptic depression, which leads to disinhibition of the P cell synapses.

In this study we examined whether endocannabinoids and N cell HFS actually depressed GABAergic input onto the P cells at the same time points that we observed potentiation of P synapses. Other studies regarding endocannabinoid-mediated disinhibition have focused on GABAergic IPSPs. Since no spontaneous IPSPs are observed in P cell recordings, we examined whether endocannabinoids mediated tonic GABAergic inhibition instead. Tonic GABAergic inhibition was observed in the P cell and endocannabinoids and N cell HFS elicited long-term depression of this tonic GABAergic input.

## Materials and Methods

### Ganglion Preparation

Medicinal leeches of the species *Hirudo verbana* (2–3g) were acquired from commercial suppliers (Leeches United States Ltd., Westbury, NY and Leech.com, Perris, California) and housed in artificial pond water (0.052% w/v Instant Ocean Sea Salt, Aquarium Systems) kept at 15°C with an alternating 12-h light/dark cycle. A single *Hirudo* would be chilled for 30 min at 4°C in pond water to lightly anesthetize the animals. Dissections of individual ganglia were conducted in ice-cold normal *Hirudo* saline solution (110 mM NaCl, 5 mM NaOH, 4 mM KCl, 1.8 mM CaCl_2_, 1 mM MgCl_2_, and 10 mM HEPES, pH 7.4) with 10 mM glucose. A single ganglion was placed in a 35 mm petri dish that had been filled with 1.5 mL Sylgard™ and has an RC-37W insert for fluid exchange (Warner Instruments; Holliston, MA, United States). Recordings were performed at room temperature (20–22°C) normal saline while maintaining a constant perfusion rate of approximately 1.5 mL/min using an eight channel valve-controlled gravity perfusion system (VC8xG) and a VWK system (ALA Scientific; Farmingdale, NY, United States). It is not necessary to oxygenate the saline during the recordings. Neurons within the ganglion and the recording electrodes were visualized using a stereomicroscope (Leica SMZ6) with magnification at 70–80× and dark-field illumination (Figure 1B).

Within the CNS of *Hirudo* is a ventral nerve cord comprised of 21 linked and almost equivalent segmental ganglia, each of which contain an estimated 400 neurons. The arrangement of these neurons is well-documented, allowing for cells to be selected for analysis based on their size, position in the ganglion, and electrophysiological properties (Muller et al., 1981). This study focused on the nociceptive (N) and pressure-sensitive (P) cells, each of which as two bilateral pairs in the ganglion. Different P cells innervate different cutaneous receptive fields, while the N cells are characterized as being either mechanical (medial N cells) or polymodal (lateral N cells) nociceptors (Nicholls and Baylor, 1968; Blackshaw et al., 1982; Pastor et al., 1996). Both pairs of N and P cells are accessible through the ventral side of the ganglion. Moreover, the P and N cells also form excitatory, glutamatergic synapses onto a number of shared targets, including the L motor neuron (shown in Figure 1A) which contributes to the *Hirudo* whole-body shortening reflex (Nicholls and Purves, 1970; Shaw and Kristan, 1995). Endocannabinoid-mediated potentiation is observed at P cell synapses onto the L motor neuron, as well as P synapses onto other postsynaptic targets (Higgins et al., 2013; Wang and Burrell, 2016).

### Drug Application

Drugs were stored in frozen aliquots and were diluted to the desired concentration in *Hirudo* saline shortly before each experiment was conducted. 2-Arachidonoyl glycerol (2-AG), *N-*arachidonylethanolamide (AEA), SB 366791, AM 251, and tetrahydrolipstatin (THL, known also as Orlistat) stocks were dissolved in dimethyl sulfoxide (DMSO). Bicuculline methiodide stocks were dissolved in normal saline. DMSO was obtained from Sigma Aldrich (St. Louis, MO, United States). 2-AG was purchased from Avanti Polar Lipids (Alabaster, AL, United States). AEA, Bicuculline (BIC), THL, and SB 366791 were acquired from Tocris/Bio-Techne (Minneapolis, MN, United States). AM 251 was obtained from Hello Bio Inc (Princeton, NJ, United States).

### Electrophysiology

Two-electrode voltage clamp (TEVC) recordings were performed using an Axoclamp 900A amplifier (Molecular Devices, San Jose, CA, United States) as in previous studies (Wang et al., 2015). Neurons were impaled with sharp glass microelectrodes directed by manual micropositioners (Model 1480, Siskiyou, Grants Pass, OR, United States). Microelectrodes were filled with a 2 M potassium acetate (KAc) solution and no KCl was included in the electrode filling solution in order to not disrupt intracellular Cl^–^ concentrations in *Hirudo* neurons, and therefore, the recordings of tonic inhibition are not from Cl^–^-loaded cells. Electrodes were fabricated from borosilicate capillary tubing (1.0-mm OD, 0.75-mm ID; FHC, Bowdoinham, ME, United States) with a horizontal puller (Sutter Instruments P-97, Novato, CA, United States). Tip resistances ranged from 35 to 50 MΩ.

P cells were voltage-clamped at −50 mV and holding current monitored using Clampex software (Molecular Devices). Following the protocols used in a variety of published studies, tonic inhibition was measured by recording holding current in normal saline and then in BIC to block tonic GABA receptor activation (Semyanov et al., 2004; Song et al., 2011; Bright and Smart, 2013). The P cell holding current was measured over an 11 min period, the first minute in normal saline followed by a 5 min application of BIC (100 μM) and then 5 min in normal saline to observe the holding current return to baseline (Figures 1C,D). For data analysis purposes, a 1 s measurement of holding current was made every 10 s for the duration of the experiment.

To observe changes in tonic GABAergic input to the P cell due to endocannabinoid treatment ganglia were treated with either 2-AG (100 μM), AEA (0.1 μM) or 0.01% DMSO (control) via bath application for 15 min. This was followed by a 30-min washout period with normal saline at which time tonic inhibition was measured to assess whether there had been a persistent effect on GABAergic input. The concentration of 2-AG used in these experiments is comparable to prior experiments using rodent brain slice preparations (Stella et al., 1997; Al-Hayani et al., 2001). In the case of these *Hirudo* studies, it should be noted that an acutely isolated ganglion is essentially an intact segment of the CNS approximately 0.5 mm thick that includes tissues (connective, muscle and glial tissue) that may impede 2-AG diffusion. In addition, this tissue does contain active MAGL that may break down a significant portion of the applied 2-AG (Kabeiseman et al., 2020).

In experiments using high-frequency stimulation (HFS) of the N cell, HFS consisted of 20 trains at a rate of 0.1 Hz, with each train having 10 suprathreshold stimulus pulses at 25 Hz (Wang and Burrell, 2016; Yuan and Burrell, 2019). Following HFS, a 30 min rest period was again used prior to testing the level of tonic inhibition. In experiments that involved application of THL (10 μM), SB 366791 (10 μM), or AM 251 (10 μM), perfusion of the recording chamber with the drug began 5 min prior to N cell HFS and continued throughout HFS period. Once the HFS was ended, the drugs were washed out for 30 min. Co-application of 2-AG and SB 366791 was conducted with the same concentrations used in earlier experiments and incubated for the same 15 min period.

### Statistics

The change in P cell holding current (pA) is reported normalized to the average of the first 6 sweeps sampled under perfusion with normal saline. All data is reported as mean ± SEM. The sample size for all experiments refers to the number of animals tested (no replicates of the same treatment in tissue from the same animal). Statistical analyses were performed with Prism GraphPad Prism 9.0 for Mac (GraphPad Software, Inc., San Diego, CA, United States). Comparisons between groups for the maximum negative displacement in holding current were performed with a one-way ANOVA followed by the Tukey’s Test for *post hoc* analysis. Results were regarded as significantly different if *p* < 0.05, <0.01, or <0.001.

## Results

**FIGURE 2:**
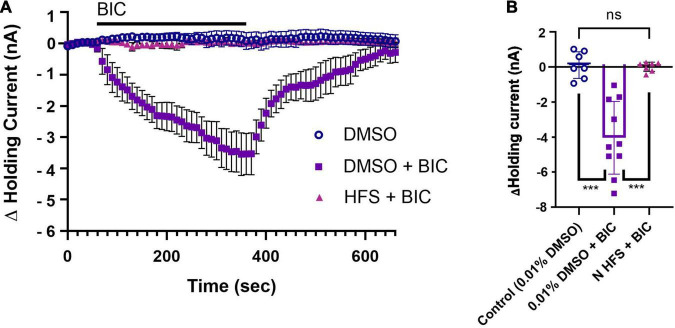
N cell HFS depresses tonic inhibition in the P cell. **(A)** A substantial shift in the P cell holding current is observed during 100 μM BIC application (DMSO + BIC group) compared to the control group that simply had the holding current monitored in vehicle control, saline with 0.01% DMSO (referred to as the DMSO group). Recall that the 0.01% DMSO in saline is applied 30 min prior to BIC application as a vehicle control experiment (see section “Materials and Methods” and Figure 1C). N cell HFS applied 30 min before BIC application (HFS + BIC) fully prevented the negative shift in P cell holding current in response to BIC and is shown to be overlain on the control group that did not receive BIC. **(B)** When comparing the peak change in holding current during BIC bath-application, changes in holding current were significantly reduced in the HFS + BIC group compared to the only DMSO + BIC group (^***^ indicates *p* < 0.0001). No difference in peak current was observed between the N cell HFS + BIC group and the DMSO group. All data is shown as mean ± SE.

The first set of experiments assessed whether P cells received tonic GABAergic input. *Hirudo* P cells have been shown to be hyperpolarized by GABA via a negative inward current and this GABA-mediated hyperpolarization/current is blocked by BIC (Sargent et al., 1977; Wang et al., 2015). In mammalian neurons, tonic inhibition has been assessed by measuring the change in holding current before and after BIC application (Semyanov et al., 2004; Song et al., 2011; Bright and Smart, 2013). This approach was used here to assess the capacity of BIC (100 μM) to elicit a similar shift in the P cell holding current, although we also looked at recovery of the holding current back to pre-treatment levels during the BIC washout phase. The downward shift in holding current during BIC application (note that these are not chloride-loaded cells) following pretreatment with normal saline containing 0.01% DMSO (DMSO + BIC group, *N* = 10) indicates that the P cell does receive tonic inhibitory input that is mediated by a GABA_*A*_-like receptor (Figure 2A). During washout from BIC, the P cell holding currents return to baseline levels. In control experiments that received the 0.01% DMSO pretreatment, but no BIC application (referred to as the DMSO group, *N* = 7), the holding current was stable throughout the entire duration of the experiment (11 min). The BIC-induced changes in the P cell holding current were significantly different from controls in which BIC was withheld (Figure 2B; one-way ANOVA *F*_2_,_21_ = 24.16, *p* < 0.0001; DMSO + BIC vs. DMSO, *post hoc p* < 0.0001).

Previous studies have shown that P cell synapses experience persistent (30–60 min), heterosynaptic potentiation following HFS of the N cells, that requires endocannabinoid signaling and GABAergic signaling (Higgins et al., 2013; Wang and Burrell, 2016). It is our hypothesis that endocannabinoids depress tonic GABAergic input to the P cell, eliciting presynaptic disinhibition that leads to this persistent synaptic potentiation (Figure 1A). The subsequent experiments are designed to test whether endocannabinoids elicited a decrease in tonic GABAergic inhibition in the P cell over the same time frame as the previously observed endocannabinoid-mediated synaptic potentiation.

Since N cell HFS elicits endocannabinoid-mediated P synapse potentiation, the first set of experiments examined the effects of such stimuli on tonic inhibition of the P cell. Thirty minutes following HFS of the N cell, the BIC-induced shift in P cell holding current was absent (Figure 2A; HFS + BIC, *N* = 7) indicating that tonic inhibition had been depressed by HFS prior to BIC application. The maximum change in holding current was significantly different between BIC application where no HFS was applied (HFS + BIC vs. DMSO + BIC, *post hoc p* < 0.0001), but not significantly different from the peak change in holding current in the no-BIC control conditions (Figure 2B; HFS + BIC vs. DMSO). To summarize, BIC application produced a negative shift in P cell holding current consistent with the presence of tonic, GABAergic inhibition. HFS of the N cell eliminated the effect of BIC on holding current indicating that this nociceptor activity depressed tonic inhibition onto the P cell.

**FIGURE 3:**
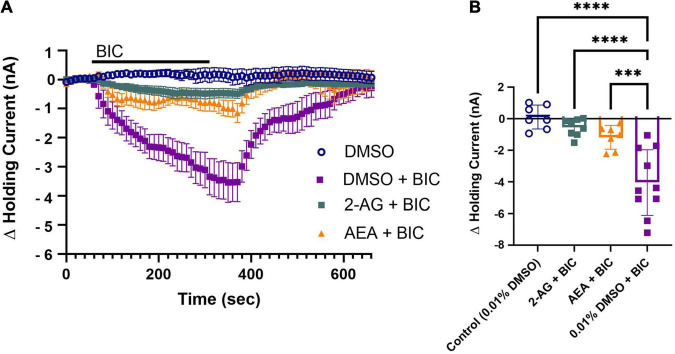
2-AG and AEA depress tonic inhibition in the P cell. **(A)** 30 min following pretreatment with 2-AG (2-AG+BIC) or AEA (AEA+BIC) produced only a very small change in holding current, indicating depression of tonic GABAergic input. **(B)** In terms of the peak change in holding current, both the 2-AG + BIC and AEA + BIC groups were significantly reduced compared to the DMSO + BIC (^***^ indicates *p* < 0.001; ^****^ indicates *p* < 0.0001). The DMSO + BIC group was also significantly different from the DMSO group. The 2-AG + BIC and AEA + BIC groups were not significantly different from the DMSO group. All data is shown as mean ± SE.

Next, the potential involvement of endocannabinoids in the depression of tonic GABAergic inhibition onto the P cell was investigated by pretreating the ganglion with either 2-AG or AEA and then monitoring the BIC-induced change in the P cell holding current after a 30 min washout period. Again, the goal here is to observe whether depression of tonic inhibition, and therefore disinhibition of the P cell, coincides with the time frame in which endocannabinoid-mediated P cell synaptic potentiation is observed. Both 2-AG and AEA are present in the *Hirudo* CNS with the concentration of 2-AG being approximately 10 times more than AEA (Matias et al., 2001). Additionally, in the rat brain, the basal levels of 2-AG have been estimated to be up to 1000 times greater than AEA (Buczynski and Parsons, 2010). Therefore, we opted to use a concentration of AEA (0.1 μM) that was 1000 times less than 2-AG (100 μM). 2-AG and AEA were applied for 15 min and then washed out for 30 min so that we could observe potential long-lasting modulatory effects initiated by either endocannabinoid. 2-AG (*N* = 8) or AEA (*N* = 7) treatment reduced BIC-induced shifts in the holding current (Figure 3A) indicating that both endocannabinoids persistently depressed tonic GABAergic signaling. The peak change in holding currents for the 2-AG- and AEA-treated P cells during BIC application was statistically lower compared to the vehicle control (DMSO group) P cell’s response to BIC (Figure 3B; *F*_3_,_28_ = 17.58, *p* < 0.0001; 2-AG + BIC vs. DMSO + BIC, *post hoc p* < 0.0001; AEA + BIC vs. DMSO + BIC, *p* < 0.0001; DMSO vs DMSO + BIC, *p* < 0.001). Although there was a small change in holding current during BIC application in the 2-AG and AEA-treated P cells (Figure 3A), no significant differences in the peak change in holding current were observed between these groups and the no-BIC control cells (Figure 3B; 2-AG + BIC vs. DMSO, AEA + BIC vs. DMSO, 2-AG + BIC vs. AEA + BIC). Thus, both endocannabinoids are able to significantly depress tonic GABA signaling onto the P cell at a time point that coincided with when 2-AG and AEA-elicited P cell synaptic potentiation was observed.

**FIGURE 4:**
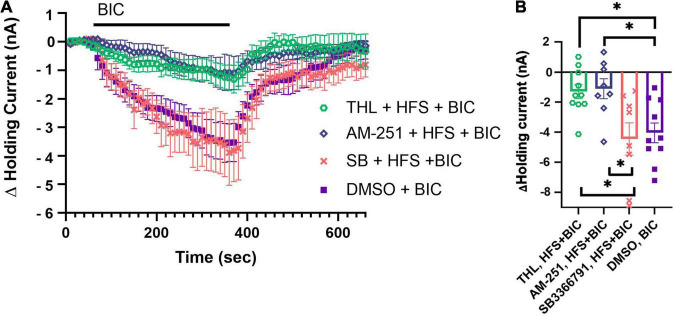
Role of DAGL, TRPV, and CB1 in mediating N cell HFS-induced depression of tonic inhibition. **(A)** Pretreatment with 10 μM SB 366791 (SB) prior to N Cell HFS restore the BIC-induced negative shift in P cell holding current. 10 μM THL/Orlistat (DAGL inhibitor) and 10 μM AM251 (CB1 reverse agonist) did not prevent N cell HFS from depressing tonic inhibition. **(B)** In terms of peak change in holding current significant differences were observed between the THL + HFS + BIC vs. the DMSO + BIC and SB + HFS + BIC groups (* indicates *p* < 0.05). Significant differences were also observed between the AM251 + HFS + BIC vs. the DMSO + BIC and SB + HFS + BIC groups. All data is shown as mean ± SE.

Since N cell HFS substantially depressed tonic inhibition onto the P cell, we next investigated whether this depression was dependent on production of 2-AG and/or TRPV receptors. This was done because both inhibition of 2-AG synthesis and TPRV channels blocked N cell HFS-induced potentiation in P synapses (Wang and Burrell, 2016). We repeated the N cell HFS experiment, but this time pretreating the ganglia with THL/Orlistat, an inhibitor of DAGL. Based on previous experiments (Yuan and Burrell, 2010, 2013b; Wang and Burrell, 2018), we hypothesized that THL/Orlistat would be able to prevent N cell HFS from depressing tonic inhibition. Following THL/Orlistat treatment, the BIC-induced change in holding current was measured 30 min following N cell HFS (*N* = 10). THL/Orlistat did not block the effects of HFS, indicated by the observations that the THL + HFS + BIC group was significantly different from the DMSO + BIC group (Figure 4; *F*_4_,_38_ = 9.29, *p* < 0.001, *post hoc p* < 0.05). Since THL/Orlistat treatment did not block the effects of N cell HFS, this suggests that 2-AG synthesis may not play a critical role in this activity-induced depression of tonic inhibition onto the P cell.

In earlier studies with *Hirudo* SB 366791, a TRPV1 receptor antagonist, blocked N cell HFS-induced potentiation of the P cell as well as potentiation produced by exogenous application of 2-AG or AEA (Higgins et al., 2013; Wang and Burrell, 2016). To determine whether a TRPV-like channel mediated N cell HFS-induced depression of tonic GABAergic input, we pretreated ganglia with SB 366791 before N cell HFS and then applied BIC 30 min later to assess tonic inhibition. SB 366791 did prevent depression of P cell tonic inhibition following N cell HFS (Figures 4A,B). The SB + HFS + BIC group (*N* = 8) was significantly different from the HFS + BIC group (*post hoc p* < 0.005) and not different from DMSO + BIC group. The peak change in holding current in the SB + HFS + BIC group was also significantly greater than the THL/Orlistat group (Figure 4B; SB + HFS + BIC vs. THL + HFS + BIC, *p* < 0.05). These findings are consistent with a TRPV-like channel playing a major role in activity-dependent depression of tonic inhibition in *Hirudo*.

The potential involvement of a *Hirudo* CB1-like metabotropic receptor was also examined. We explored the ability of AM251, an inverse agonist of CB1 receptors, to block the N cell HFS-induced depression of tonic inhibition onto the P cell. Ganglia were pre-treated with AM251 followed by N cell HFS and then BIC application 30 min later. While a small change in the in holding current was observed in the AM 251-treated ganglia (Figure 4A, *N* = 8), the peak shift in P cell holding current in the AM251 + HFS + BIC was not statistically different from the HFS + BIC group. The AM251 + HFS + BIC group was statistically different from the DMSO + BIC and the SB + HFS + BIC groups administered to control ganglia (Figure 4B, *p* < 0.05 for both). These results suggest that a CB1-like receptor does not contribute to HFS-induced depression of tonic inhibition.

**FIGURE 5:**
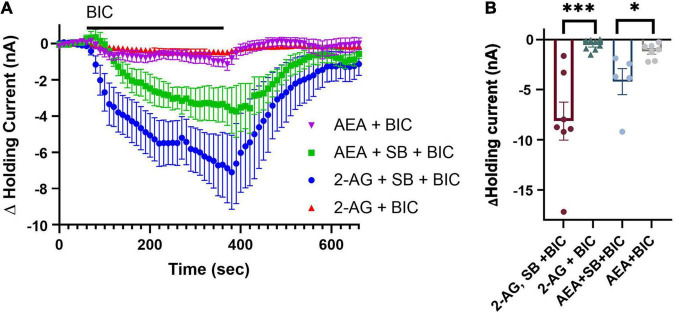
TPRV signaling mediates 2-AG- and AEA-induced depression of tonic inhibition. **(A)** Co-application of 100 μM 2-AG with 10 μM SB is able to prevent 2-AG-induced depression of tonic inhibition onto the P cell. A total of 10 μM SB was also able to block the 0.1 μM AEA-induced depression of tonic inhibition. **(B)** In terms of peak current, the 2-AG + SB + BIC group was significantly different from the 2-AG + BIC group while the AEA + SB + BIC group was significantly different from the AEA + BIC group (* indicates *p* < 0.05; *** indicates *p* < 0.001). All data is shown as mean ± SE.

Finally, we examined whether SB 366791 would block 2-AG- and AEA-mediated depression of tonic inhibition of the P cell observed in Figure 3. Ganglia were pre-treated with SB 366791 followed by SB 366791 plus 2-AG or AEA for 15 min. This was followed 30 min later by BIC application during which the P cell holding current was recorded. In the 2-AG + SB group (*N* = 8), BIC was now able to elicit a change in holding current, indicating that TRPV blocker did prevent depression of tonic inhibition by 2-AG (Figures 5A,B; 2-AG + BIC vs. SB + 2-AG + BIC, unpaired *t*-test t_13_ = 4.29, *p* < 0.0001). In the AEA + SB group (*N* = 5), BIC was also able to elicit a change in holding current, indicating that SB 366791 prevented AEA-induced depression of tonic inhibition (Figures 5A,B; 2-AG + BIC vs. SB + 2-AG + BIC, unpaired *t*-test t_13_ = 2.68, *p* < 0.05). These results indicate that depression of tonic inhibition by either 2-AG or AEA is mediated by a *Hirudo* TRPV-like channels.

## Discussion

In this study we observed endocannabinoid-mediated long-term depression (LTD) of GABAergic input. However, instead of LTD of GABAergic IPSPs as previously reported (Zhu and Lovinger, 2007; Gibson et al., 2008), we observed depression of tonic GABAergic inhibition. The pressure sensitive (P) mechanosensory cells in the *Hirudo* CNS exhibit tonic GABAergic inhibition based on changes in holding current during BIC application similar to how tonic inhibition has been observed in a variety of mammalian neurons (Semyanov et al., 2004; Song et al., 2011; Bright and Smart, 2013). The source of this putative GABAergic input is not known, but the *Hirudo* CNS does possess GABA-containing neurons that are continuously active (Cline, 1983, 1986). Tonic inhibition has been shown to be an important regulator of excitability and synaptic transmission in both the brain and spinal cord of mice and rats (Semyanov et al., 2004; Pavlov et al., 2014; Gradwell et al., 2017). In *Hirudo*, exogenous application of GABA does hyperpolarize the P cells and disinhibition via BIC application enhances P cell synaptic transmission at the presynaptic level (Wang et al., 2015). This disinhibition plays a critical role in endocannabinoid-mediated potentiation of *Hirudo* P cell synapses. Application of either 2-AG or AEA produces persistent (at least 30–60 min) potentiation in P synapses that is prevented by co-application of BIC or inhibitors of Cl^–^ transport (Higgins et al., 2013; Wang and Burrell, 2016). HFS of a single *Hirudo* nociceptor (N cell) elicits a heterosynaptic form of synaptic potentiation in P synapses that is 2-AG-mediated (prevented by inhibition of the DAGL inhibitor, THL) and also prevented by blocking disinhibition (Wang and Burrell, 2016).

A lack of change in the holding current following BIC application is indicative that tonic GABAergic input is depressed. In the *Hirudo* P cells, BIC produced little or no change in holding current following 2-AG or AEA treatment, an indication that both endocannabinoids had depressed tonic inhibition to the P cell. This effect was long-lasting and the depression of tonic inhibition coincided with the time when endocannabinoid-mediated disinhibition/potentiation was observed in the previous experiments (Higgins et al., 2013; Wang and Burrell, 2016). The effects of 2-AG and AEA on tonic inhibition were blocked by the TRPV1 inhibitor SB66791. TRPV1 is known to be an important cannabinoid receptor in mammals for both AEA and 2-AG (Zygmunt et al., 1999, 2013; Gibson et al., 2008; De Petrocellis et al., 2011). TRPV-encoding genes are present in *Hirudo* (Heath-Heckman et al., 2021) and pharmacological studies using both activators (capsaicin and resiniferatoxin) and inhibitors (capsazepine and SB366791) support that a TRPV-like channel acts as a cannabinoid receptor in this species (Yuan and Burrell, 2010; Li and Burrell, 2011; Higgins et al., 2013; Wang and Burrell, 2016).

In the present study, it is proposed that depression of tonic inhibition is mediated by endocannabinoids acting on TRPV-like channels located on GABAergic interneurons (Figure 1A). In studies of endocannabinoid-mediated LTD of nociceptive (N) synapses in *Hirudo*, which are glutamatergic, intracellular injection of two different TRPV inhibitors (capsazepine and SB366791) demonstrated that a TRPV-like channel was acting presynaptically (Yuan and Burrell, 2010, 2013b). This endocannabinoid-mediated LTD of N synapses is mediated by activation of presynaptic calcineurin and coordinated transcription and translation in the pre- and postsynaptic neurons (Yuan and Burrell, 2012, 2013a). We hypothesize that similar cellular mechanisms are involved in endocannabinoid-mediated depression of tonic inhibition although additional experiments will be needed to confirm this. The identity of the GABAergic neurons undergoing depression is unknown so one cannot definitively prove the involvement of presynaptic TRPV-like channels as was done for the N synapse. However, the P cells, which receive this tonic inhibitory input, lack TRPV-like channels themselves (Summers et al., 2014). Therefore, the GABAergic neurons represent the most likely location for the TRPV-like channels mediating depression of tonic inhibition.

TRPV channels have been found to be required for endocannabinoid-mediated LTD via both presynaptic (Gibson et al., 2008) and postsynaptic mechanisms (Chávez et al., 2010; Grueter et al., 2010) in rodents. It may seem surprising that TRPV channels, which conduct Ca^2+^, could mediate LTD presynaptically since increases in presynaptic Ca^2+^ would be expected to enhance neurotransmitter release and there are multiple reports of increases in spontaneous EPSP frequency during TRPV1 activation (Baccei et al., 2003; Medvedeva et al., 2008; Peters et al., 2010; Park et al., 2011). How then can presynaptic TRPV channels mediate synaptic depression? One potential explanation is that while TRPV activation can enhance synaptic transmission in the short-term during the period of direct activation, the resulting Ca^2+^ influx can also trigger intracellular signaling cascades that act over longer time frames in which depression is observed (tens of minutes to hours). This is analogous to NMDA-type glutamate receptors which can contribute to an EPSP, but also produce LTP or LTD through Ca^2+^-dependent activation of intracellular signaling processes. Our studies of endocannabinoid-mediated depression of excitatory synapses and tonic inhibition have all been conducted with a considerable delay/washout period between drug application or HFS/LFS and the actual measurement of synaptic transmission. Furthermore, our past studies have shown that synaptic transmission following endocannabinoid activation of TRPV requires increases in intracellular Ca^2+^, activation of calcineurin, and new protein synthesis, all at the presynaptic level (Yuan and Burrell, 2012, 2013a). These findings are consistent with a long-lasting modulatory process.

Another factor to consider is that there is a discontinuity in the effects of TRPV activation on spontaneous EPSPs vs. evoked EPSPs. The studies of TRPV1’s presynaptic facilitating effects all involved increases in frequency of spontaneous EPSPs. However, one of these studies also reported that capsaicin depressed evoked EPSPs at the same time that spontaneous EPSP frequency was increased (Medvedeva et al., 2008) and there are additional reports of TRPV1 activation resulting in synaptic depression that is mediated presynaptically (Yang et al., 1999; Kusudo et al., 2006). As a possible explanation for how presynaptic TRPV could have opposing effects on spontaneous vs. evoked synaptic transmission, Fawley et al. (2016) have reported that there are distinct Ca^2+^ sources or nano-domains contributing to the spontaneous vs. evoked neurotransmitter release. In their experiments, TRPV1 channels could stimulate spontaneous neurotransmitter release, without contributing to evoked release.

N cell HFS also depressed tonic inhibition to the P cell. We investigated the role of 2-AG mediating this effect by blocking the activity of the DAGL, the primary enzyme involved in 2-AG synthesis (Lee et al., 1995). In previous experiments, THL/Orlistat blocked potentiation of P synapses following N cell HFS (Wang and Burrell, 2016), suggesting 2-AG involvement. Surprisingly, inhibition of 2-AG synthesis did not prevent the effects of N cell HFS on tonic inhibition. While there was a small shift in holding current during BIC application in the THL + HFS + BIC group, this was not significantly different from the HFS + BIC group. This would suggest that even though 2-AG can directly depress tonic inhibition, 2-AG is not required for HFS-induced depression. Perhaps AEA is mediating HFS-induced depression of tonic inhibition, while 2-AG plays some other, unknown role in mediating endocannabinoid-dependent LTP. Future experiments are planned to investigate the role of AEA in more detail.

The TRPV1 channel inhibitor, SB 366791, completely blocked depression of the tonic inhibition following N cell HFS. As noted above, a TRPV-like channel is thought to be a cannabinoid receptor in *Hirudo* and the TRPV inhibitor SB 366791 has also been observed to block HFS-elicited heterosynaptic potentiation of P synapses (Wang and Burrell, 2016). AM 251, a CB1 receptor reverse agonist, did not affect depression of tonic inhibition following N cell HFS. To date, the earliest homolog to the CB1 and CB2 receptors has been observed in *Ciona*, a deuterostome invertebrate (Egertova and Elphick, 2007; Elphick, 2012). Recently however, an endocannabinoid-sensitive metabotropic receptor has been identified in *C. elegans*, a protostome invertebrate (Pastuhov et al., 2016; Oakes et al., 2017). In addition, AM 251 has been found to inhibit putative endocannabinoid modulation in a number of different protostome invertebrates, including *Hirudo* (Lemak et al., 2007; Li and Burrell, 2009; Sunada et al., 2017). It remains to be determined whether these previously observed effects of AM 251 on invertebrates are due to action on a CB1 receptor ortholog or perhaps an alternative cannabinoid-sensitive metabotropic receptor (Paulsen and Burrell, 2019).

To our knowledge, this is the first report of activity-dependent modulation of tonic inhibition and endocannabinoid-mediated depression of tonic GABAergic inhibition. There is considerable evidence of the cannabinoid system being able to modulate activity-dependent synaptic plasticity, in particular LTP (Piette et al., 2020). In many cases, endocannabinoids reduce inhibitory synaptic transmission, thereby lowering the threshold for eliciting LTP (Chevaleyre and Castillo, 2004; Zhu and Lovinger, 2007; Xu et al., 2012), often referred to as metaplasticity (Abraham and Bear, 1996). Tonic inhibition has also been identified as an important modulator of LTP, again by altering the threshold for LTP induction (Martin et al., 2010) or enhancing one form of LTP over another (Dembitskaya et al., 2020). In our previous studies, N cell HFS alone is able to elicit potentiation of P synapses, without the need for the P cell to be directly activated. It is possible that during N cell HFS, endocannabinoid-mediated depression of tonic inhibition in the P cell creates a state where spontaneous/background activity is sufficient to elicit an LTP-like process (see Figure 1A). A comparable mechanism has been suggested in which high enough levels of 2-AG promote spike-timing dependent LTP without the need for coordinated pre- and postsynaptic activity in the rat striatum (Cui et al., 2016). Similarly, application of tetrahydrocannabinol alone has been reported to elicit widespread LTP in the mouse hippocampus (Puighermanal et al., 2009).

What is the functional relevance of this observed depression of tonic inhibition following HFS of nociceptors and its proposed contribution to potentiation of P synapses? Potentiation of *Hirudo* P synapses is observed in semi-intact preparations following either HFS of a single N cell or delivery of a noxious stimulus to the skin (Wang and Burrell, 2018). This synaptic potentiation is endocannabinoid- and TRPV-dependent and contributes to behavioral sensitization in *Hirudo* in reflexive withdrawal responses to P cell stimulation. These findings in *Hirudo* have relevance to nociception in other species. Both synaptic and tonic inhibition play an important role in regulating activity in nociceptive circuits and disinhibition of these circuits contributes to sensitization to nociceptive and non-nociceptive stimuli following injury in rodents (Torsney and MacDermott, 2006; Kim et al., 2012; Petitjean et al., 2015; Gradwell et al., 2017; Perez-Sanchez et al., 2017). We propose that strong nociceptor activation elicits endocannabinoid synthesis and release (Figure 1A). These endocannabinoids, in turn, mediate heterosynaptic depression of inhibitory input with the resulting disinhibition contributing to behavioral sensitization. This hypothesis is supported by both our findings in *Hirudo* (Higgins et al., 2013; Wang and Burrell, 2016, 2018) and those by Pernia-Andrade et al. (2009) in mice. While the latter study involved activation of CB1, another study has shown that TRPV channel activation in the mouse spinal cord can also mediate disinhibition that contributes to allodynia (Kim et al., 2012). This study did not identify the ligand activating TRPV1, but endocannabinoids are a potential candidate.

Activity-dependent depression of tonic inhibition, potentially via endocannabinoids, represents an important, but until now unrecognized, modulatory process that can contribute to plasticity in synaptic circuits. From a functional standpoint, such modulation of tonic inhibition can contribute not only to nociception, but also other neurobehavioral process, e.g., learning memory. These findings are also relevant to understanding endocannabinoids’ dual nature in synaptic modulation. That is, their ability to potentiate synapses under some conditions (often, but not exclusively via disinhibition) and depress synapses under other conditions (i.e., depression of neurotransmitter release). Examining how different patterns of activity and different intracellular signaling cascades produce this weakening or strengthening of synapses represents the critical next steps in understanding the endocannabinoid system’s role in neurobehavioral plasticity.

## Data Availability Statement

The raw data supporting the conclusions of this article will be made available by the authors, without undue reservation.

## Author Contributions

RP and BB developed the experimental design, carried out the experiments, analyzed the data, and wrote the manuscript. Both authors contributed to the article and approved the submitted version.

## Conflict of Interest

The authors declare that the research was conducted in the absence of any commercial or financial relationships that could be construed as a potential conflict of interest.

## Publisher’s Note

All claims expressed in this article are solely those of the authors and do not necessarily represent those of their affiliated organizations, or those of the publisher, the editors and the reviewers. Any product that may be evaluated in this article, or claim that may be made by its manufacturer, is not guaranteed or endorsed by the publisher.
